# Paradoxical Lower Serum Triglyceride Levels and Higher Type 2 Diabetes Mellitus Susceptibility in Obese Individuals with the *PNPLA3* 148M Variant

**DOI:** 10.1371/journal.pone.0039362

**Published:** 2012-06-18

**Authors:** Colin N. A. Palmer, Cristina Maglio, Carlo Pirazzi, Maria Antonella Burza, Martin Adiels, Lindsay Burch, Louise A. Donnelly, Helen Colhoun, Alexander S. Doney, John F. Dillon, Ewan R. Pearson, Mark McCarthy, Andrew T. Hattersley, Tim Frayling, Andrew D. Morris, Markku Peltonen, Per-Arne Svensson, Peter Jacobson, Jan Borén, Lars Sjöström, Lena M. S. Carlsson, Stefano Romeo

**Affiliations:** 1 Medical Research Institute, Ninewells Hospital and Medical School, University of Dundee, Dundee, Scotland, United Kingdom; 2 Department of Molecular and Clinical Medicine and Center for Cardiovascular and Metabolic Research, Sahlgrenska Academy, University of Gothenburg, Gothenburg, Sweden; 3 Oxford Centre for Diabetes, Endocrinology and Metabolism, University of Oxford, Oxford, United Kingdom; 4 Peninsula NIHR Clinical Research Facility, Peninsula College of Medicine and Dentistry, University of Exeter, Exeter, United Kingdom; 5 Chronic Disease Epidemiology and Prevention Unit, Department of Chronic Disease Prevention, National Institute for Health and Welfare, Helsinki, Finland; 6 Department of Clinical and Experimental Medicine, University of Catanzaro, Catanzaro, Italy; University of Tübingen, Germany

## Abstract

**Background:**

Obesity is highly associated with elevated serum triglycerides, hepatic steatosis and type 2 diabetes (T2D). The I148M *(rs738409*) genetic variant of patatin-like phospholipase domain-containing 3 gene (*PNPLA3)* is known to modulate hepatic triglyceride accumulation, leading to steatosis. No association between *PNPLA3* I148M genotype and T2D in Europeans has been reported. Aim of this study is to examine the relationship between *PNPLA3* I148M genotypes and serum triglycerides, insulin resistance and T2D susceptibility by testing a gene-environment interaction model with severe obesity.

**Methods and Findings:**

*PNPLA3* I148M was genotyped in a large obese cohort, the SOS study (n = 3,473) and in the Go-DARTS (n = 15,448), a T2D case-control study. Metabolic parameters were examined across the *PNPLA3* I148M genotypes in participants of the SOS study at baseline and at 2- and 10-year follow up after bariatric surgery or conventional therapy. The associations with metabolic parameters were validated in the Go-DARTS study. Serum triglycerides were found to be lower in the *PNPLA3* 148M carriers from the SOS study at baseline and from the Go-DARTS T2D cohort. An increased risk for T2D conferred by the 148M allele was found in the SOS study (O.R. 1.09, 95% C.I. 1.01-1.39, P = 0.040) and in severely obese individuals in the Go-DARTS study (O.R. 1.37, 95% C.I. 1.13-1.66, P = 0.001). The 148M allele was no longer associated with insulin resistance or T2D after bariatric surgery in the SOS study and no association with the 148M allele was observed in the less obese (BMI<35) individuals in the Go-DARTS study (P for interaction  = 0.002). This provides evidence for the obesity interaction with I48M allele and T2D risk in a large-scale cross-sectional and a prospective interventional study.

**Conclusions:**

Severely obese individuals carrying the *PNPLA3* 148M allele have lower serum triglyceride levels, are more insulin resistant and more susceptible to T2D. This study supports the hypothesis that obesity-driven hepatic lipid accumulation may contribute to T2D susceptibility.

## Introduction

Hepatic steatosis, serum triglycerides, insulin resistance and type 2 diabetes are tightly associated [1]–[5]. The most widely replicated genetic variant associated with hepatic steatosis is an isoleucine to methionine substitution at position 148 (I148M, rs738409) in the patatin-like phospholipase domain-containing 3 gene (*PNPLA3)* [6]–[11]. To date, no association between *PNPLA3* I148M genotype and type 2 diabetes in Europeans has been reported [9], [12]–[14]. However, a recent study showed that hepatic Pnpla3 overexpression leads to changes in the glucose and triglycerides homeostasis in mouse models of obesity and diabetes [15]. Moreover, genetic reports in Europeans indicate that obesity modifies signals arising from the genome, showing that the *PNPLA3* I148M genotype association with increased alanine transferase (ALT) is greatly exacerbated in obesity [12], [16], [17].

To test a gene-environment interaction model with severe obesity in determining metabolic traits, we examined the relationship between *PNPLA3* I148M genotypes, serum triglycerides, insulin resistance and type 2 diabetes susceptibility in a large obese cohort, the Swedish Obese Subjects (SOS) study, before and after bariatric surgery. We also validated our findings in the Genetics of Diabetes Audit and Research Tayside Scotland (Go-DARTS) study, a type 2 diabetes case-control study.

## Methods

### Study design

**Figure 1:**
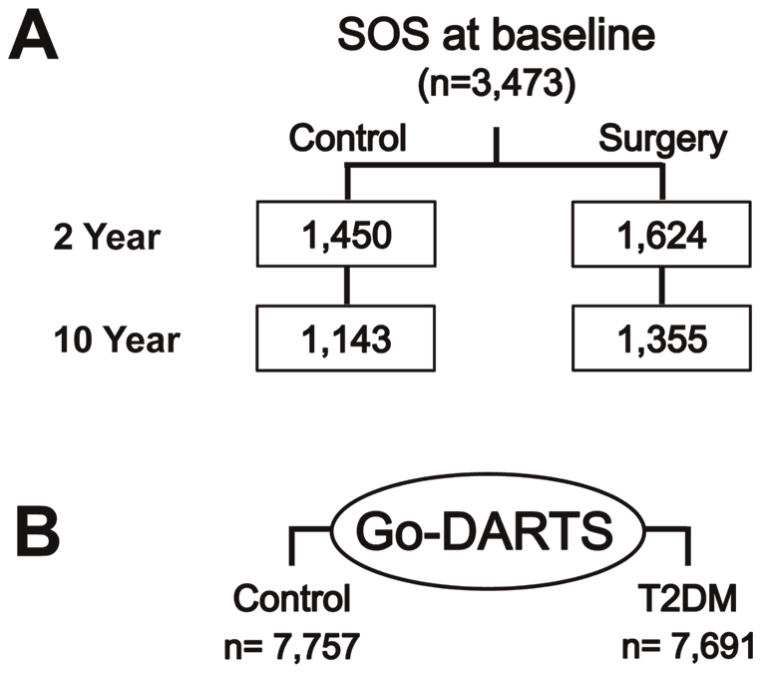
Study design. *PNPLA3* I148M was genotyped in the Swedish Obese Subjects (SOS) study (A) and Genetics of Diabetes Audit and Research Tayside Scotland (Go-DARTS) study (B) participants (n = 3,473 and 15,448 respectively).

This genetic study involved two independent European cohorts: the SOS and the Go-DARTS studies (Figure 1). The SOS is a prospective controlled intervention trial that compares the long term effects of bariatric surgery and conventional care in obese subjects (Figure 1A). The Go-DARTS is a cross sectional population case control study for type 2 diabetes comprising normal weight to morbidly obese individuals (Figure 1B).

The hypothesis of an association between *PNPLA3* I148M genotype and serum triglycerides, insulin resistance (assessed by homeostasis model assessment for insulin resistance, HOMA-IR) and type 2 diabetes was tested in obese participants of the SOS study before any treatment (baseline, Figure 1A). Subsequently, the associations found at baseline were investigated at 2- and 10-year follow up by analyzing separately the control (individuals who did not have substantial changes in body weight), and the surgery group (individuals who underwent bariatric surgery) to test if the associations were preserved at different time points and if they were affected by severe weight loss.

Next, the Go-DARTS study (Figure 1B) participants were examined to confirm the SOS study findings in an independent study cohort. Specifically, the *PNPLA3* I148M association with serum triglycerides and type 2 diabetes risk in severely obese individuals (Body-mass index ≥35) was tested.

### Ethic statement

Written Informed consent has been obtained by all study participants. All clinical investigations have been conducted according to the principles expressed in the Declaration of Helsinki. The SOS study protocol was approved the following Swedish ethics committees: Regional Institutional Review Board of Gothenburg University, Regional Institutional Review Board of Linköping University, Regional Institutional Review Board of Lund University, Regional Institutional Review Board of Karolinska Intitute, in Stockholm, Regional Institutional Review Board of Umeå University, Regional Institutional Review Board of Örebro University and Regional Institutional Review Board of Uppsala University. SOS trial has been registered in the ClinicalTrials.gov registry (NCT01479452, http://clinicaltrials.gov/ct2/show/NCT01479452?term  =  NCT01479452&rank  = 1). The Go-DARTS study has been approved by the Tayside Medical ethics Committee at the Ninewells Hospital and Medical School, Dundee, UK.

### The SOS study

The SOS study has been previously described [18], [19]. Briefly, the study enrolled 4,047 obese individuals in Sweden between September 1987 and January 2001. A total of 2,010 individuals constituted the bariatric surgery arm and a matched control group of 2,037 individuals was enrolled based on 18 matching variables. Inclusion and exclusion criteria have been presented previously [18]. A total of 3,585 DNA samples from SOS study participants (SOS Version 1.0) was available for analyses. *PNPLA3* was successfully genotyped in 3,473 individuals: 1,719 from the control group and 1,754 from the surgery group (success rate: 97%).

The surgery patients and the conventionally treated controls both started the study 4 weeks before the date of bariatric surgery (termed baseline). Biochemical parameters were measured at baseline and after 2 and 10 years in both the control and the surgery group. All blood samples were obtained in fasting conditions [18], [19]. Type 2 diabetes (n = 521 at baseline) was classified as fasting blood glucose ≥6.1 mmol/L (corresponding to fasting plasma glucose ≥7.0 mmol/L or 126 mg/dL) and/or therapy with glucose-lowering medications. At each examination, weight, height and blood pressure were measured.

### The Go-DARTS study

The Go-DARTS study is a Wellcome Trust-sponsored ongoing cohort study examining the genetic factors that contribute to type 2 diabetes and related conditions [20]–[22]. Recruitment was performed in the Tayside region of Scotland. All participants are of European ancestry. Between 1997 and 2009, Go-DARTS recruited 9,000 individuals with type 2 diabetes and 8,187 individuals without type 2 diabetes. *PNPLA3* I148M genotyping was available for a total of 7,691 and 7,757 individuals in the case and control group, respectively.

Retrospective and follow up data are ascertained from the Tayside region electronic medical record. Biochemical parameters were measured at the study visit. ALT levels were available from routine clinical database.

### Genotyping of the *PNPLA3* I148M genetic variant (rs738409)

Fluorogenic 5′-nucleotidase (Taqman) allelic discrimination assay for the *PNPLA3* rs738409 sequence variant was used to genotype all participants, as previously described [12], [16], with a success rate of 97%. To ensure for the quality and consistency of genotyping, three positive controls (one for each PNPLA3 rs738409 I148M genotype) which were previously genotyped by direct Sanger sequencing were included in each plate of the allelic discrimination assay. Furthermore, each DNA sample was run in triplicate and genotype calls with a 100% consistency were included in the analyses.

### Statistical analyses

Variables are shown as means ± standard deviations. HOMA-IR was calculated only in non-diabetic participants as: [fasting insulin mIU/L x fasting glucose mmol/L]/22.5 [23]. Genotype and allele as well as categorical variable distributions across the genotype classes were compared using χ^2^ test. Linear regression analysis was used to assess the effect of the three different *PNPLA3* genotypes on continuous dependent variables and P values were adjusted for age, gender and body-mass index (BMI). P values were similar after adjustment of triglycerides for HOMA-IR and ALT or after adjustment of HOMA-IR for ALT and triglycerides. Changes at 2- and 10- year follow up have been calculated as follows: [(follow up value – baseline value)/ baseline value] ×100. Binary logistic regression analysis was used to calculate allelic odds ratio and 95% confidence interval for insulin resistance, type 2 diabetes and combined risk; age, gender, and BMI were included in the model as covariates.

Non-normally distributed variables were log transformed before entering the statistical models. Statistical analyses were carried out using the Statistical Package for Social Sciences (SPSS, version 18.0.0, Inc. Chicago, IL, USA). P values <0.05 were considered significant.

## Results

### *PNPLA3* 148M allele is associated with metabolic traits in obese subjects in the SOS study before bariatric surgery

To assess the effect of the *PNPLA3* I148M genotype on serum lipids and insulin resistance in obese subjects, DNA from SOS participants was genotyped (Figure 1A). Clinical characteristics of participants at baseline (n = 3,473; combined control and surgery groups) are shown in Table S1. Genotype and allele frequencies in these subjects were in Hardy-Weinberg equilibrium (Table S2) and they were not different from those previously observed in Europeans [24]. The clinical characteristics at baseline stratified for *PNPLA3* I148M genotypes are presented in Table 1. All participants were severely obese at baseline (mean BMI 41±5, Table S1). As a positive control for the genetic analyses ALT were examined. Increased serum levels of ALT were found in carriers of the 148M allele at baseline (P<0.001; Table 1). Triglycerides were found to be lower (P<0.001; Table 1) and HOMA-IR (P = 0.004; Table 1) to be higher in the *PNPLA3* 148M carriers at baseline.

**Table 1:** Baseline Clinical Characteristics of SOS Study Participants Stratified by *PNPLA3* I148M Genotype.

	PNPLA3 genotype			
Characteristic	II	IM	MM	P Value*
*N*	2,139	1,179	155	-
Male (%)	30	30	26	0.509
Age (years)	48±6	48±6	48±7	0.924
Body-mass index	41±5	41±5	42±4	0.139
Systolic blood pressure (mmHg)	141±18	142±19	139±20	0.954
Diastolic blood pressure (mmHg)	88±11	88±11	87±11	0.682
Glucose (mg/dL)	90±33	93±37	90±40	0.092
Insulin (mIU/L)†	18±10	19±12	19±11	0.002
HOMA-IR†	3.6±2.2	3.9±2.7	3.9±2.3	0.004
Total cholesterol (mg/dL)	223±43	220±41	216±39	0.020
HDL cholesterol (mg/dL)	52±12	52±13	51±11	0.582
Triglycerides (mg/dL)	193±134	190±135	167±80	<0.001
AST (IU/L)	23±11	26±16	29±13	<0.001
ALT (IU/L)	34±21	39±30	45±28	<0.001
Alcohol intake (g/week)	5±8	5±8	5±7	0.951
Lipid-lowering medications (%)	2	2	0	0.077
Type 2 diabetes (%)	14	17	12	0.046

Abbreviations: SOS, Swedish obese subjects; PNPLA3, patatin-like phospholipase domain-containing 3; II, individuals with two 148I alleles; MM, individuals with two 148M alleles; IM, heterozygotes; n, number; HOMA-IR, homeostasis model assessment for insulin resistance; HDL, high-density lipoprotein; AST, aspartate transferase; ALT, alanine transferase.

Plus-minus values are means ± SD.

* P values were calculated using linear regression model including age, gender and body-mass index for all variables. HOMA-IR, triglycerides, ALT and AST were log-transformed before entering the model. Male gender, lipid-lowering medications and type 2 diabetes distribution were compared by χ2 test. See methods for more details on the statistical analyses.

† Fasting insulin and HOMA-IR are shown only in non-diabetic individuals.

Next the risk of being insulin resistant was examined in non diabetic individuals at baseline. The individuals were divided into insulin resistant and sensitive according to the median of the HOMA-IR (in men 3.9 and in women 2.9 U). The *PNPLA3* 148M variant was associated with a higher risk of being insulin resistant (Odds ratio, O.R.: 1.10, 95% Confidence interval, C.I. 1.02-1.35, P = 0.038; Panel A, Table S3). Next, the risk of type 2 diabetes was examined and the *PNPLA3* 148M allele was also associated with a significantly higher risk of type 2 diabetes at baseline after adjusting for age, gender, and BMI (O.R. 1.09, 95% C.I. 1.01-1.39, P = 0.040, Panel B, Table S3). The combined risk of developing insulin resistance or diabetes was consistently increased (O.R. 1.11, 95% C.I. 1.03-1.37, P = 0.021, Panel C, Table S3).

### Associations between *PNPLA3* 148M allele and metabolic traits are abolished by surgically induced weight loss

**Figure 2:**
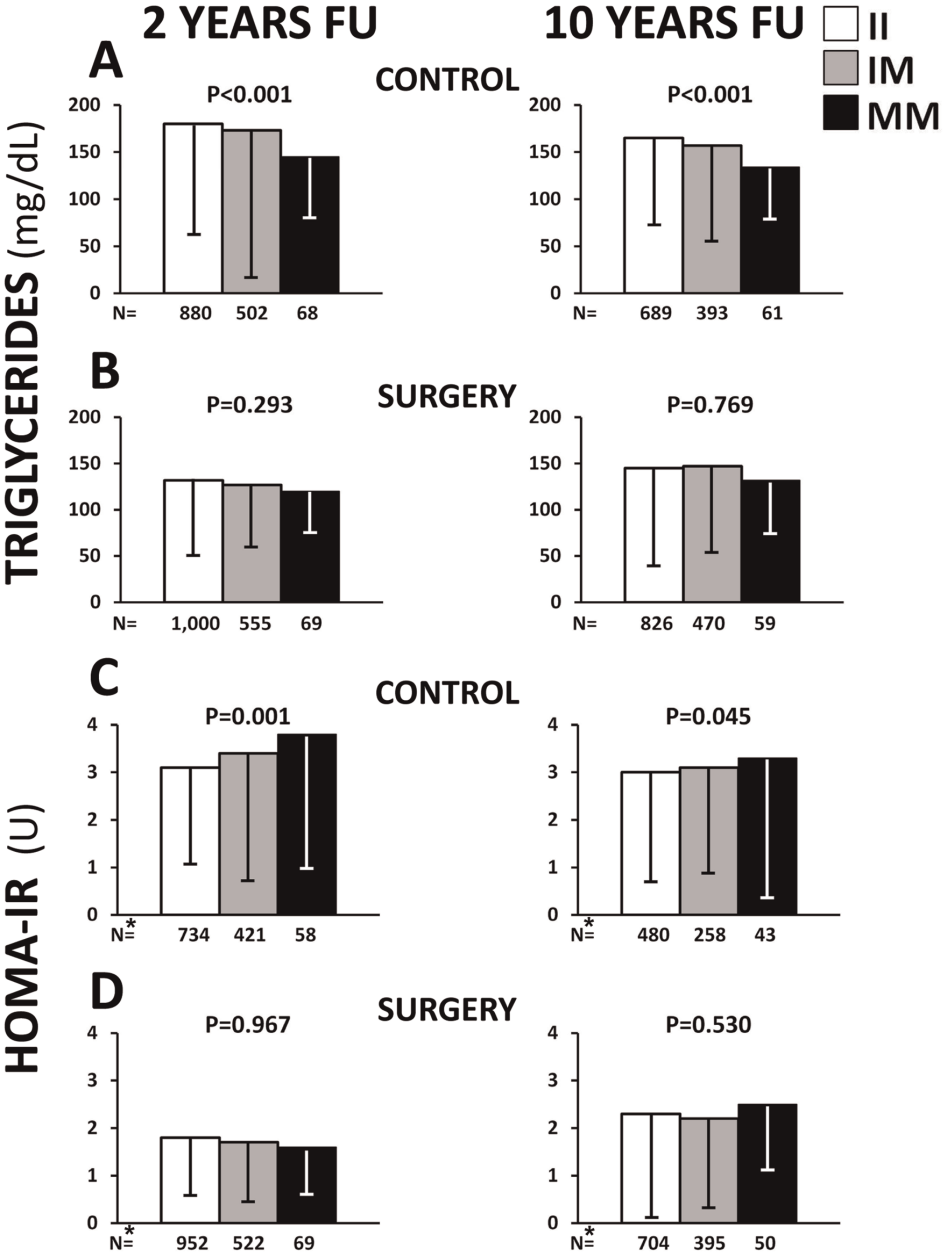
Serum triglycerides and homeostasis model assessment for insulin resistance (HOMA-IR) at 2- and 10-year follow-up. Serum triglycerides (panel A and B) levels and HOMA-IR (panel C and D) at 2- and 10- year follow up (FU) in the control (A, C) and the surgery (B, D) group across the *PNPLA3* genotypes. Values are means and standard deviations. * HOMA-IR values are shown only in non-diabetic individuals.

The clinical characteristics at 2- and 10- year follow up in the control and the surgery group separately, stratified for *PNPLA3* I148M genotypes are shown in table S4 and S5, respectively. Weight changes in the surgery and the control group from baseline to 10-year follow up have been previously described [19]. Briefly, there was almost no weight loss in the control group at the 2- and 10-year follow up, whereas the mean BMI in the surgery group was reduced by 24% at 2- year and 17% at 10- year follow up. Serum triglycerides were lower in *PNPLA3* 148M carriers in the control at 2- and 10-year follow up (P<0.001 in both, Figure 2A) but not in the surgery group (Figure 2B). HOMA-IR was greater in carriers of the 148M allele in the control group at the 2-year (P = 0.001) and 10-year (P = 0.045) follow up (Figure 2C), but no differences were found across *PNPLA3* genotypes in the surgery group (Figure 2D). In the 2- and 10- year follow up there was no difference in the *PNPLA3* I148M genotype distribution in participants with and without type 2 diabetes in both the control and surgery group (Table S4 and S5).

As a positive control for the genetic analyses, ALT levels were examined. Increased serum levels of ALT were found in carriers of the 148M allele in the control group at the 2-year (P<0.001) and 10-year (P = 0.004) follow up (Table S4). In the surgery group no differences in ALT levels were found across *PNPLA3* genotypes (Table S5) at 2- year follow up whereas at 10-year follow up ALT increased across the *PNPLA3* genotypes (P = 0.002; table S5).

Moreover, 2- and 10- year changes in metabolic parameters were tested across the genotype classes. In the surgery group, the *PNPLA3* 148M allele was associated with a higher reduction in HOMA-IR and ALT levels and with a lower reduction in triglyceride levels at 2- and 10-year follow up (Table S6). No associations were observed in the individuals without surgery.

### Association of *PNPLA3* 148M Allele with Metabolic Traits in the Go-DARTS Study

The effect of the *PNPLA3* 148M allele on metabolic traits was also examined in the Go-DARTS study, a large case (n = 7,691) control (n = 7,757) study of type 2 diabetes based in the Tayside region of Scotland (for clinical characteristics Table S7). Genotype and allele frequencies were in Hardy-Weinberg Equilibrium (Table S8). Lower levels of fasting serum triglycerides coupled with an increase in ALT levels (both P<0.001) were observed in 148M diabetic carriers but not in the control group (Table 2). Subsequently, the Go-DARTS diabetic participants with BMI ≥35 (n = 1,842) and <35 (n = 5,849) were analyzed separately. The association with ALT levels was present in both groups (data not shown), but the association with serum triglycerides was only found in the severely obese (BMI≥35) group (II: 225±131, IM: 208±132, MM: 191±131 mg/dL, P = 0.003) and not in those with BMI<35 (II: 190±122, IM: 184±117, MM: 184±120 mg/dL, P = 0.219), although the interaction term did not reach statistical significance (P = 0.18). After stratifying the Go-DARTS control group by BMI, no associations between serum triglyceride levels and *PNPLA3* were found in individuals either with BMI≥35 or <35. However, it should be pointed out that the severely obese group without type 2 diabetes was small (n = 456).

**Table 2:** Clinical Characteristics of Go-DARTS Study Participants Stratified by PNPLA3 I148M Genotype.

	Type 2 Diabetes				Control			
	PNPLA3 genotype				PNPLA3 genotype			
Characteristic	II	IM	MM	P Value*	II	IM	MM	P Value*
*N*	4,798	2,553	340	-	4,947	2,476	334	-
Age (years)	66±11	66±11	66±12	ns	61±13	61±13	61±13	ns
Body-mass index	31±6	32±6	31±6	ns	27±5	27±4	27±4	ns
Systolic blood pressure (mmHg)	142±19	142±19	140±18	ns	136±20	136±20	135±18	ns
Diastolic blood pressure (mmHg)	76±11	76±11	77±12	ns	80±10	79±10	79±10	ns
Glucose (mg/dL)	-	-	-	-	89±15	89±11	90±14	ns
Insulin (mIU/L)	-	-	-	-	11±8	11±7	10±9	ns
HOMA-IR	-	-	-	-	1.7±1.4	1.7±1.2	1.7±1.7	ns
Total cholesterol (mg/dL)	169±36	168±37	169±36	ns	205±41	204±41	203±41	ns
HDL cholesterol (mg/dL)	52±15	52±15	51±14	ns	63±18	63±18	62±17	ns
Triglycerides (mg/dL)	198±132	189±114	188±111	<0.001	138±87	139±98	132±72	ns
ALT (IU/L)	31±27	34±25	42±30	<0.001	27±34	30±70	28±17	ns
HbA1c (%)	7.5±1.4	7.5±1.5	7.4±1.4	ns	5.5±0.4	5.5±0.4	5.5±0.5	ns

Abbreviations: Go-DARTS, Genetics of Diabetes Audit and Research Tayside Scotland; PNPLA3, patatin-like phospholipase domain-containing 3; II, individuals with two 148I alleles; MM, individuals with two 148M alleles; IM, heterozygotes; n, number; HOMA-IR, homeostasis model assessment for insulin resistance HDL, high-density lipoprotein; ALT, alanine transferase; HbA1c, glycated hemoglobin; ns, P value≥0.05.

Plus-minus values are means ± SD.

* P values were calculated using linear regression model including age, body-mass index and gender for all variables. HOMA-IR, triglycerides and ALT were log-transformed before entering the model. See methods for more details on the statistical analyses.

Moreover, the *PNPLA3* 148M allele was associated with a marginally increased risk of developing type 2 diabetes in the overall study (O.R. 1.04, 95% C.I. 0.98-1.11, P = 0.104, table 3A). After stratifying the cohort according to BMI, the 148M allele was found to confer a significantly increased risk in those with BMI ≥35 (n = 1,842 in the type 2 diabetes group and n = 456 in the control group; O.R. 1.37, 95% C.I. 1.13-1.66, P = 0.001, table 3B) but not in those with BMI <35 (n = 5,849 in the type 2 diabetes group and n = 7,301 in the control group; O.R. 1.01, 95% C.I. 0.94-1.07, P = 0.855, table 3B). A highly significant statistical interaction term between *PNPLA3* genotype and severe obesity status was observed in determining risk of type 2 diabetes (P = 0.002).

**Table 3:** Multivariate regression analysis of Risk of Type 2 Diabetes Mellitus in the Go-DARTS study.

**A: Unstratified analysis**				
			**Confidence Interval**	
	**P value** *	**OR**	**Lower**	**Upper**
Gender (F)	<0.0001	0.73	0.68	0.79
Age	<0.0001	1.05	1.05	1.05
BMI	<0.0001	1.19	1.18	1.20
PNPLA3 148M allele	0.104	1.04	0.98	1.11
**B: Stratified by Severe Obesity**				
	**BMI<35**			
			**Confidence Interval**	
	**P value** *	**OR**	**Lower**	**Upper**
Gender (F)	<0.0001	0.77	0.71	0.83
Age	<0.0001	1.05	1.05	1.06
BMI	<0.0001	1.20	1.19	1.21
PNPLA3 148M allele	0.855	1.01	0.94	1.07
	**BMI≥35**			
			**Confidence Interval**	
	**P value** *	**OR**	**Lower**	**Upper**
Gender (F)	<0.0001	0.56	0.45	0.70
Age	<0.0001	1.02	1.01	1.03
BMI	<0.0001	1.10	1.07	1.14
PNPLA3 148M allele	0.001	1.37	1.13	1.66

* P-values were calculated under an additive model using a binary logistic regression model including gender as a categorical variable (Males  =  referent) and age and BMI as continuous variables.

P value for interaction between severe obesity and I148M for type 2 diabetes risk  = 0.002.

Abbreviations: Go-DARTS, Genetics of Diabetes Audit and Research Tayside Scotland; OR, odds ratio; F, female; BMI, body mass index; PNPLA3, patatin-like phospholipase domain-containing 3.

## Discussion

This is the first study showing that the *PNPLA3* 148M allele is associated with insulin resistance and increased type 2 diabetes risk specifically in severely obese individuals despite relatively lower serum triglycerides. The results of this study are supported by extensive prior research that has established the robust gene/environment interaction between the *PNPLA3* 148M allele and obesity in determining individual susceptibility to hepatocyte lipid accumulation and damage [10], [12], [16], [24]. This interaction is clearly evident in our study, as the obese *PNPLA3* 148M carriers in the SOS study had higher ALT levels before, but not after, weight loss caused by bariatric surgery. We have now shown that the association of the *PNPLA3* 148M variant with other phenotypes is also dependent on increased body mass *per se* or on factors related to it.

Lower serum triglycerides and higher insulin resistance in 148M carriers were found before but not after surgery. This difference in insulin resistance was seen in the individuals without diabetes but was also reflected in an observed 15% increased risk for type 2 diabetes in the pre-surgery SOS study participants for each *PNPLA3* 148M allele. The absence of the association with type 2 diabetes in the SOS control group at 2- and 10- year follow up is likely due to a lack of statistical power given the reduced sample size.

Furthermore, we examined if *PNPLA3* I148M variant affects changes in insulin resistance, triglyceride and transaminase levels. In the surgery group, the *PNPLA3* 148M allele was associated with a higher reduction in HOMA-IR and ALT levels and with a lower reduction in triglyceride levels at 2- and 10-year follow up. No associations were present in the control group. This result reflects the association of *PNPLA3* with HOMA-IR, triglyceride and ALT levels before but not after weight loss.

Similar observations were made in the Go-DARTS study, where the association with triglycerides was dependant on obesity, with the lean group showing no association. The association with diabetes was also consistent between the two studies. While the overall increased risk of the *PNPLA3* 148M allele for type 2 diabetes in the Go-DARTS study was only 4%, in the severely obese a 37% increased risk per 148M allele for type 2 diabetes was found. Again, no association with type 2 diabetes was seen in those with a lower BMI.

The mean BMI of the Go-DARTS diabetic cohort is similar to that of the SOS surgery group in the follow up. However, no effect of the *PNPLA3* genotype on serum parameters was observed in the SOS surgery group. Surgery may lead to a great improvement of the metabolic profile due to profound reductions in calorific intake and thus blunt the effect of the *PNPLA3* genotype. Indeed, despite the similar BMI, the SOS surgery group shows an overall healthier metabolic profile compared to the Go-DARTS diabetic cohort.

The gene/obesity interaction, where the association is only seen in a fairly extreme subgroup, may explain why the *PNPLA3* 148M allele has not been widely associated with serum triglyceride levels, insulin resistance and type 2 diabetes in other studies [6], [24], [25]. In many population-based or healthy volunteer studies, the prevalence of severe obesity would be rather limited. Moreover, a low statistical power may explain the lack of significant associations in previous studies performed on obese subjects [10], [12], [16]. To date, two well-powered genetic studies have shown an association of the *PNPLA3* 148M allele with lower fasting serum triglycerides; this association was specifically present in overweight/obese individuals and no association was found in the other non-obese cohorts [24], [26]. Furthermore, a recent genetic report showed an association of the 148M allele with insulin resistance in individuals of Asian descent with NAFLD where the authors raised the question regarding the possibility of an association of the 148M allele and diabetes mellitus [27], however this study was confounded by the fact that the cases were ascertained by hepatic steatosis status, therefore the causal relationships between the genotype and insulin resistance could not be established.

The cut off point of a BMI of 35 used in the analysis of the Go-DARTS cohort was selected to match the severe obesity in the SOS study, but further analysis showed that this was an optimal split point with the associations with serum triglycerides and type 2 diabetes being limited to individuals with severe obesity. In fact, none of these associations were apparent in the overweight or moderately obese groups (25.1–34.9, data not shown), although the association between 148M and ALT levels was only absent in individuals with a BMI <25 in the Go-DARTS cohort, presumably reflecting the more pronounced gene environment interaction of this variant and hepatocyte damage. This notion is supported by the fact that only the association with ALT levels was apparent upon the 10 year weight rebound in the SOS surgery group.

The novelty of our analysis resides not only in the *PNPLA3* 148M allele being associated with type 2 diabetes but also simultaneously with lower serum triglycerides and increased insulin resistance. Furthermore, it supports a recent study in which serum triglyceride raising alleles were not found associated with increased risk for type 2 diabetes [28]. In line with this, the GCKR gene sequence variant (rs780094) has been found consistently associated with increased serum triglycerides, decreased insulin resistance and protection from type 2 diabetes [13], [29], [30]. Moreover, results of this study also suggest the possibility of finding new genetic loci associated with type 2 diabetes, insulin resistance or fasting triglycerides levels uncovered by obesity which would serve as a stressor acting on the genetic background. *In vivo* genetically modified murine models often reveal glucose or lipid metabolism changes after obesity is induced by genetic manipulation or high fat diet [31].

Although no effect on lipid or glucose metabolism was found in the *Pnpla3* knock out mouse model [32], [33], a recent study of mice overexpressing hepatic Pnpla3 demonstrated marked changes in serum triglycerides and glucose [15]. Fasting serum triglycerides are a reflection of the basal hepatic VLDL efflux as opposed to postprandial serum triglycerides which consist mainly of chylomicrons [34]. PNPLA3 is a membrane bound protein highly expressed in hepatocytes [35]. It is tightly associated with the endoplasmic reticulum and lipid droplets [36] where nascent VLDL are enriched in triglycerides. This protein shows an in vitro hydrolytic lipase activity [36] on triglycerides which may be responsible for the nascent VLDL lipidation. The PNPLA3 148M mutant protein fails to hydrolyze triglycerides in vitro [36]. The loss of function mutation could account for a reduction of the VLDL lipidation and a subsequent impaired hepatic triglycerides efflux resulting in lower serum triglycerides and liver fat retention. Under these circumstances liver fat accumulation may lead to insulin resistance and possibly to type 2 diabetes in obese individuals. Further physiological studies are warranted to understand the exact role of PNPLA3 in mediating the observed phenotypes.

A strength of this study is the evaluation of two large, well-powered and phenotyped European study cohorts and the use of a surgical intervention to prospectively modify the gene/obesity interaction within individuals. A limitation of this study is the lack of imaging data regarding the accumulation of lipids in the livers of the obese subjects; however the role of PNPLA3 148M in modulating hepatic triglyceride accumulation and the corresponding relationships with altered ALT values is well established by many previous studies. Another limitation of this study is the use of HOMA-IR to assess insulin resistance; studies on selected severe obese individuals using euglycaemic clamp to measure insulin resistance are needed to confirm the association of the 148M allele and insulin resistance.

In conclusion, this study demonstrates a replicated gene by obesity interaction in determining type 2 diabetes risk, and provides insight into the causal role of obesity-driven hepatic lipid accumulation and type 2 diabetes.

## Supporting Information

Table S1 **Clinical Characteristics of SOS Study Participants at Baseline.**
(DOC)

Table S2 **Genotype and Allele Frequencies of the** ***PNPLA3*** **I148M Sequence Variant** (**rs738409**) **in the SOS Study Participants.**
(DOC)

Table S3 **Multivariate regression analysis of insulin resistance, type 2 diabetes and combined risk in the SOS study.**
(DOC)

Table S4 **Clinical Characteristics of SOS Study Control Group Stratified by** ***PNPLA3*** **I148M Genotype at 2- and 10-Year Follow Up.**
(DOC)

Table S5 **Clinical Characteristics of SOS Study Surgery Group Stratified by** ***PNPLA3*** **I148M Genotype at 2- and 10-Year Follow Up.**
(DOC)

Table S6 **Two- and ten-year changes in HOMA-IR, triglyceride and ALT values in the surgery and the control group from the SOS study.**
(DOC)

Table S7 **Clinical Characteristics of Go-DARTS Study Participants.**
(DOC)

Table S8 **Genotype and Allele Frequencies of the PNPLA3 I148M Sequence Variant in the Type 2 Diabetes and Control Groups from the Go-DARTS Study.**
(DOC)
