# Supplementary material for: Paradoxical Lower Serum Triglyceride Levels and Higher Type 2 Diabetes Mellitus Susceptibility in Obese Individuals with the PNPLA3 148M Variant
Source: PLoS One. 2012 Jun 18;7(6):e39362. doi: 10.1371/journal.pone.0039362 (PMC3377675; doi:10.1371/journal.pone.0039362)
Supplement: Table S1 — Clinical Characteristics of SOS Study Participants at Baseline. (DOC) [file pone.0039362.s001.doc]

**Table S1.** Clinical Characteristics of SOS Study Participants at Baseline.

| *Characteristic* | |
| --- | --- |
| *n* | 3,473 |
| Surgery group (%) | 51 |
| Male (%) | 30 |
| Age (years) | 48±6 |
| Body-mass index | 41±5 |
| Systolic blood pressure (mmHg) | 141±19 |
| Diastolic blood pressure (mmHg) | 88±11 |
| Glucose (mg/dL) | 91±35 |
| Insulin (mIU/L) * | 18±11 |
| HOMA-IR * | 3.7±2.4 |
| Type 2 diabetes (%) | 15 |
| Total cholesterol (mg/dL) | 222±42 |
| HDL cholesterol (mg/dL) | 52±12 |
| Triglycerides (mg/dL) | 191±133 |
| AST (IU/L) | 25±13 |
| ALT (IU/L) | 36±25 |
| Alcohol intake (g/week) | 37±55 |
| Lipid-lowering medications (%) | 2 |
| Glucose-lowering medications (%) | 8 |

Abbreviations: SOS, Swedish obese subjects; n, number; HOMA-IR, homeostasis model assessment for insulin resistance; HDL, high-density lipoprotein; AST, aspartate transferase; ALT, alanine transferase.

Plus-minus values are means ±SD.

*Fasting insulin and HOMA-IR are shown only in non-diabetic individuals.
