# Supplementary material for: Paradoxical Lower Serum Triglyceride Levels and Higher Type 2 Diabetes Mellitus Susceptibility in Obese Individuals with the PNPLA3 148M Variant
Source: PLoS One. 2012 Jun 18;7(6):e39362. doi: 10.1371/journal.pone.0039362 (PMC3377675; doi:10.1371/journal.pone.0039362)
Supplement: Table S2 — Genotype and Allele Frequencies of the PNPLA3 I148M Sequence Variant (rs738409) in the SOS Study Participants. (DOC) [file pone.0039362.s002.doc]

**Table S2.** Genotype and Allele Frequencies of the *PNPLA3* I148M Sequence Variant (rs738409) in the SOS Study Participants.

|  | **Overall** | **No Diabetes** | **Type 2 Diabetes** |
| --- | --- | --- | --- |
| II (%) | 2,139 (62)* | 1,830 (62) | 303 (58)^§^ |
| IM (%) | 1,179 (34) | 976 (33) | 200 (38) |
| MM (%) | 155 (4) | 137 (5) | 18 (4) |
| Total | 3,473 | 2,943 | 521 |
|  |  |  |  |
| I (%) | 5,457 (79) | 4,636 (79) | 806 (77) |
| M (%) | 1,489 (21) | 1,250 (21) | 236 (23) |
| Total | 6,946 | 5,886 | 1,042 |

Abbreviations: PNPLA3, patatin-like phospholipase domain-containing 3; SOS, Swedish obese subjects; II, individuals with two 148I alleles; MM, individuals with two 148M alleles; IM, heterozygotes.

* Genotype distribution is in Hardy-Weinberg equilibrium.

§ No diabetes vs diabetes, P=0.046 with χ^2^ test.
