# Supplementary material for: Paradoxical Lower Serum Triglyceride Levels and Higher Type 2 Diabetes Mellitus Susceptibility in Obese Individuals with the PNPLA3 148M Variant
Source: PLoS One. 2012 Jun 18;7(6):e39362. doi: 10.1371/journal.pone.0039362 (PMC3377675; doi:10.1371/journal.pone.0039362)
Supplement: Table S3 — Multivariate regression analysis of insulin resistance, type 2 diabetes and combined risk in the SOS study. (DOC) [file pone.0039362.s003.doc]

**Table S3.** Multivariate regression analysis of insulin resistance, type 2 diabetes and combined risk in the SOS study.

|  | 1. **Insulin resistance** | | | | 1. **Type 2 diabetes** | | | | 1. **Combined*** | | | |
| --- | --- | --- | --- | --- | --- | --- | --- | --- | --- | --- | --- | --- |
|  |  |  | **Confidence Interval** | |  |  | **Confidence Interval** | |  |  | **Confidence Interval** | |
|  | **P value** | **OR** | **Lower** | **Upper** | **P value** | **OR** | **Lower** | **Upper** | **P value** | **OR** | **Lower** | **Upper** |
| Gender (F) | 0.003 | 0.77 | 0.65 | 0.91 | <0.001 | 0.49 | 0.40 | 0.60 | <0.001 | 0.65 | 0.56 | 0.77 |
| Age | 0.054 | 1.01 | 1.00 | 1.03 | <0.001 | 1.05 | 1.03 | 1.07 | <0.001 | 1.02 | 1.01 | 1.04 |
| BMI | <0.001 | 1.15 | 1.13 | 1.17 | 0.058 | 1.02 | 0.99 | 1.04 | <0.001 | 1.13 | 1.11 | 1.15 |
| PNPLA3 148M allele | 0.038 | 1.10 | 1.02 | 1.35 | 0.040 | 1.09 | 1.01 | 1.39 | 0.021 | 1.11 | 1.03 | 1.37 |

Abbreviations: SOS, Swedish obese subjects; OR, odds ratio; F, female; BMI, body mass index; PNPLA3, patatin-like phospholipase domain-containing 3.

P-values were calculated using a binary logistic regression model including gender as a categorical variable (Males = referent) and age and BMI as continuous variables. Odd ratio per copy of M allele is shown.

*It refers to the combined risk of insulin resistance and type 2 diabetes.
