# Supplementary material for: Paradoxical Lower Serum Triglyceride Levels and Higher Type 2 Diabetes Mellitus Susceptibility in Obese Individuals with the PNPLA3 148M Variant
Source: PLoS One. 2012 Jun 18;7(6):e39362. doi: 10.1371/journal.pone.0039362 (PMC3377675; doi:10.1371/journal.pone.0039362)
Supplement: Table S4 — Clinical Characteristics of SOS Study Control Group Stratified by PNPLA3 I148M Genotype at 2- and 10-Year Follow Up. (DOC) [file pone.0039362.s004.doc]

**Table S4.** Clinical Characteristics of SOS Study Control Group Stratified by *PNPLA3* I148M Genotype at 2- and 10-Year Follow Up.

|  | **2-Year Follow Up** | | | | **10-Year Follow Up** | | | |
| --- | --- | --- | --- | --- | --- | --- | --- | --- |
|  | **PNPLA3 genotype** | | |  | **PNPLA3 genotype** | | |  |
| **Characteristic** | **II** | **IM** | **MM** | **P Value*** | **II** | **IM** | **MM** | **P Value*** |
| *n* | 880 | 502 | 68 | - | 689 | 393 | 61 | **-** |
| Male (%) | 30 | 32 | 25 | 0.381 | 29 | 33 | 28 | 0.487 |
| Age (years) | 51±6 | 51±6 | 51±7 | 0.864 | 58±6 | 59±6 | 59±7 | 0.295 |
| Body-mass index | 40±5 | 40±5 | 40±4 | 0.267 | 40±6 | 40±6 | 40±6 | 0.817 |
| Systolic blood pressure (mmHg) | 137±17 | 137±17 | 137±18 | 0.805 | 140±17 | 140±18 | 143±18 | 0.606 |
| Diastolic blood pressure (mmHg) | 84±10 | 84±11 | 84±10 | 0.898 | 83±9 | 83±10 | 84±10 | 0.665 |
| Glucose (mg/dL) | 90±33 | 90±35 | 93±41 | 0.633 | 100±40 | 98±36 | 101±45 | 0.466 |
| Insulin (mIU/L)**†** | 16±9 | 17±13 | 19±12 | 0.001 | 15±10 | 16±10 | 16±12 | 0.026 |
| HOMA-IR**†** | 3.1±2.0 | 3.4±2.8 | 3.8±2.9 | 0.001 | 3.0±2.4 | 3.1±2.2 | 3.3±2.7 | 0.045 |
| Total cholesterol (mg/dL) | 216±41 | 211±40 | 206±37 | 0.004 | 204±40 | 199±40 | 205±35 | 0.158 |
| HDL cholesterol (mg/dL) | 53±14 | 53±13 | 54±11 | 0.965 | 53±14 | 53±14 | 55±13 | 0.295 |
| Triglycerides (mg/dL) | 180±120 | 173±160 | 145±62 | <0.001 | 165±89 | 157±101 | 134±52 | <0.001 |
| AST (IU/L) | 24±10 | 25±13 | 28±15 | <0.001 | 27±13 | 28±10 | 31±14 | 0.001 |
| ALT (IU/L) | 32±19 | 35±21 | 45±34 | <0.001 | 31±22 | 33±18 | 36±25 | 0.004 |
| Alcohol intake (g/week) | 5±10 | 6±9 | 4±6 | 0.694 | 5±9 | 6±10 | 4±6 | 0.949 |
| Lipid-lowering medications (%) | 4 | 2 | 2 | 0.200 | 17 | 18 | 10 | 0.300 |
| Type 2 diabetes (%) | 16 | 16 | 13 | 0.803 | 30 | 34 | 30 | 0.364 |

Abbreviations: SOS, Swedish obese subjects; PNPLA3, patatin-like phospholipase domain-containing 3; II, individuals with two 148I alleles; MM, individuals with two 148M alleles; IM, heterozygotes; n, number; HOMA-IR, homeostasis model assessment for insulin resistance HDL, high-density lipoprotein; AST, aspartate transferase; ALT, alanine transferase.

Plus-minus values are means ±SD.

*P values were calculated using linear regression model including age, gender and body-mass index for all variables. HOMA-IR, triglycerides, ALT and AST and were log-transformed before entering the model. Male gender, lipid-lowering medications and type 2 diabetes distribution were compared by χ^2^ test. See methods for more details on the statistical analyses.

† Fasting insulin and HOMA-IR are shown only in non-diabetic individuals.
