# Supplementary material for: Paradoxical Lower Serum Triglyceride Levels and Higher Type 2 Diabetes Mellitus Susceptibility in Obese Individuals with the PNPLA3 148M Variant
Source: PLoS One. 2012 Jun 18;7(6):e39362. doi: 10.1371/journal.pone.0039362 (PMC3377675; doi:10.1371/journal.pone.0039362)
Supplement: Table S5 — Clinical Characteristics of SOS Study Surgery Group Stratified by PNPLA3 I148M Genotype at 2- and 10-Year Follow Up. (DOC) [file pone.0039362.s005.doc]

**Table S5.** Clinical Characteristics of SOS Study Surgery Group Stratified by *PNPLA3* I148M Genotype at 2- and 10-Year Follow Up.

|  | **2-Year Follow Up** | | | | **10-Year Follow Up** | | | |
| --- | --- | --- | --- | --- | --- | --- | --- | --- |
|  | **PNPLA3 genotype** | | |  | **PNPLA3 genotype** | | |  |
| **Characteristic** | **II** | **IM** | **MM** | **P Value*** | **II** | **IM** | **MM** | **P Value*** |
| *n* | 1,000 | 555 | 69 | - | 826 | 470 | 59 | - |
| Male (%) | 31 | 29 | 28 | 0.625 | 29 | 31 | 25 | 0.660 |
| Age (years) | 49±6 | 49±6 | 49±6 | 0.619 | 57±6 | 57±6 | 58±6 | 0.967 |
| Body-mass index | 32±5 | 32±5 | 32±5 | 0.492 | 35±6 | 35±6 | 35±5 | 0.671 |
| Systolic blood pressure (mmHg) | 135±19 | 136±20 | 134±19 | 0.342 | 141±19 | 141±18 | 136±17 | 0.517 |
| Diastolic blood pressure (mmHg) | 83±11 | 84±11 | 83±11 | 0.217 | 85±10 | 85±11 | 84±11 | 0.518 |
| Glucose (mg/dL) | 76±20 | 75±17 | 72±9 | 0.182 | 84±28 | 85±32 | 81±16 | 0.821 |
| Insulin (mIU/L)**†** | 10±6 | 10±6 | 9±5 | 0.775 | 12±10 | 11±8 | 13±7 | 0.506 |
| HOMA-IR**†** | 1.8±1.3 | 1.7±1.2 | 1.6±0.8 | 0.967 | 2.3±2.2 | 2.2±1.8 | 2.5±1.4 | 0.530 |
| Total cholesterol (mg/dL) | 218±43 | 215±39 | 215±47 | 0.202 | 211±42 | 212±43 | 212±41 | 0.799 |
| HDL cholesterol (mg/dL) | 63±15 | 62±15 | 61±14 | 0.277 | 61±17 | 61±16 | 58±12 | 0.132 |
| Triglycerides (mg/dL) | 132±80 | 127±68 | 120±47 | 0.293 | 145±111 | 147±86 | 132±54 | 0.769 |
| AST (IU/L) | 21±18 | 21±8 | 22±15 | 0.602 | 26±10 | 28±14 | 30±14 | <0.001 |
| ALT (IU/L) | 24±27 | 22±14 | 23±18 | 0.262 | 25±15 | 28±17 | 30±20 | 0.002 |
| Alcohol intake (g/week) | 36±57 | 41±95 | 38±52 | 0.183 | 44±87 | 46±74 | 30±44 | 0.582 |
| Lipid-lowering medications (%) | 2 | 1 | 1 | 0.869 | 12 | 10 | 12 | 0.687 |
| Type 2 diabetes (%) | 5 | 5 | 0 | 0.146 | 14 | 15 | 12 | 0.807 |

Abbreviations: SOS, Swedish obese subjects; PNPLA3, patatin-like phospholipase domain-containing 3; II, individuals with two 148I alleles; MM, individuals with two 148M alleles; IM, heterozygotes; n, number; HOMA-IR, homeostasis model assessment for insulin resistance; HDL, high-density lipoprotein; AST, aspartate transferase; ALT, alanine transferase;

Plus-minus values are means ±SD.

*P values were calculated using linear regression model including age, gender and body-mass index for all variables. HOMA-IR, triglycerides, ALT and AST were log-transformed before entering the model. Male gender, lipid-lowering medications and type 2 diabetes distribution were compared by χ^2^ test. See methods for more details on the statistical analyses.

†Fasting insulin and HOMA-IR are shown only in non-diabetic individuals.
