# Supplementary material for: Paradoxical Lower Serum Triglyceride Levels and Higher Type 2 Diabetes Mellitus Susceptibility in Obese Individuals with the PNPLA3 148M Variant
Source: PLoS One. 2012 Jun 18;7(6):e39362. doi: 10.1371/journal.pone.0039362 (PMC3377675; doi:10.1371/journal.pone.0039362)
Supplement: Table S6 — Two- and ten-year changes in HOMA-IR, triglyceride and ALT values in the surgery and the control group from the SOS study. (DOC) [file pone.0039362.s006.doc]

**Table S6.** Two- and ten-year changes in HOMA-IR, triglyceride and ALT values in the surgery and the control group from the SOS study.

|  | **METABOLIC CHANGES (%**) | | **PNPLA3 genotype** | | |  |
| --- | --- | --- | --- | --- | --- | --- |
|  |  |  | **II** | **IM** | **MM** | **P value*** |
| **SOS Control Group** | HOMA-IR | 2-year **†** | 12±62 | 8±47 | 13±53 | 0.233 |
|  |  | 10-year **†** | 24±68 | 15±65 | 20±78 | 0.612 |
|  | Triglycerides | 2-year | 5±42 | 7±70 | -4±25 | 0.450 |
|  |  | 10-year | 3±45 | 3±46 | -5±38 | 0.259 |
|  | ALT | 2-year | 9±50 | 8±48 | 11±48 | 0.812 |
|  |  | 10-year | 14±57 | 9±58 | -8±63 | 0.333 |
| **SOS Surgery Group** | HOMA-IR | 2-year **†** | -49±39 | -54±35 | -59±30 | 0.024 |
|  |  | 10-year **†** | -28±24 | -32±28 | -34±33 | 0.047 |
|  | Triglycerides | 2-year | -29±20 | -27±19 | -25±20 | 0.010 |
|  |  | 10-year | -19±15 | -18±20 | -16±31 | 0.042 |
|  | ALT | 2-year | -23±20 | -35±36 | -45±42 | <0.001 |
|  |  | 10-year | -19±28 | -22±15 | -25±50 | 0.068 |

Abbreviations: HOMA-IR, homeostasis model assessment for insulin resistance; ALT, alanine transferase; SOS, Swedish obese subjects; PNPLA3, patatin-like phospholipase domain-containing 3; II, individuals with two 148I alleles; MM, individuals with two 148M alleles; IM, heterozygotes.

† HOMA-IR changes are shown only in non-diabetic individuals.

*****P values were calculated using linear regression model including age, gender and body-mass index for all variables**.**

Plus-minus values are means ±SD.
