# Supplementary material for: Paradoxical Lower Serum Triglyceride Levels and Higher Type 2 Diabetes Mellitus Susceptibility in Obese Individuals with the PNPLA3 148M Variant
Source: PLoS One. 2012 Jun 18;7(6):e39362. doi: 10.1371/journal.pone.0039362 (PMC3377675; doi:10.1371/journal.pone.0039362)
Supplement: Table S7 — Clinical Characteristics of Go-DARTS Study Participants. (DOC) [file pone.0039362.s007.doc]

**Table S7.** Clinical Characteristics of Go-DARTS Study Participants**.**

| **Characteristic** | **Type 2 Diabetes** | **Control** | **P Value*** |
| --- | --- | --- | --- |
| n | 7,691 | 7,757 | - |
| Male (%) | 56 | 50 | <0.001 |
| Age (years) | 66±11 | 61±13 | 0.010 |
| Body-mass index | 31±6 | 27±5 | <0.001 |
| Systolic blood pressure (mmHg) | 142±19 | 136±19 | 0.017 |
| Diastolic blood pressure (mmHg) | 76±11 | 79±10 | <0.001 |
| Total cholesterol (mg/dL) | 169±36 | 204±41 | <0.001 |
| HDL cholesterol (mg/dL) | 52±15 | 63±18 | <0.001 |
| Triglycerides (mg/dL) | 194±125 | 138±89 | <0.001 |
| ALT (IU/L) | 33±26 | 28±48 | <0.001 |
| HbA1c (%) | 7.4±1.4 | 5.5±0.4 | <0.001 |
| Glucose-lowering medications (%) | 75 | 0 | <0.001 |
| Lipid-lowering medications (%) | 79 | 25 | <0.001 |

Abbreviations: Go-DARTS, Genetics of Diabetes Audit and Research Tayside Scotland; n, number; HDL, high-density lipoprotein; ALT, alanine transferase; HbA1c, glycated hemoglobin.

Plus-minus values are means ±SD.

*P values were calculated using linear regression model including age, body-mass index and gender for all variables. Triglycerides and ALT were log-transformed before entering the model. Male gender, glucose and lipid-lowering medications distribution were compared by χ^2^ test.
