# Supplementary material for: Paradoxical Lower Serum Triglyceride Levels and Higher Type 2 Diabetes Mellitus Susceptibility in Obese Individuals with the PNPLA3 148M Variant
Source: PLoS One. 2012 Jun 18;7(6):e39362. doi: 10.1371/journal.pone.0039362 (PMC3377675; doi:10.1371/journal.pone.0039362)
Supplement: Table S8 — Genotype and Allele Frequencies of the PNPLA3 I148M Sequence Variant in the Type 2 Diabetes and Control Groups from the Go-DARTS Study. (DOC) [file pone.0039362.s008.doc]

**Table S8.** Genotype and Allele Frequencies of the PNPLA3 I148M Sequence Variant in the Type 2 Diabetes and Control Groups from the Go-DARTS Study.

|  | **Type 2 Diabetes** | **Control** | **P Value†** |
| --- | --- | --- | --- |
| **Overall** |  |  |  |
| II (%) | 4,798 (63)* | 4,947 (64)* |  |
| IM (%) | 2,553 (33) | 2,476 (32) |  |
| MM (%) | 340 (4) | 334 (4) |  |
| Total | 7,691 | 7,757 | 0.103 |
|  |  |  |  |
| I (%) | 12,152 (79) | 12,365 (80) |  |
| M (%) | 3,230 (21) | 3,149 (20) |  |
| Total | 15,382 | 15,514 | 0.132 |
| **BMI< 35** |  |  |  |
| II (%) | 3,688 (63) | 4,638 (64) |  |
| IM (%) | 1,899 (33) | 2,344 (32) |  |
| MM (%) | 262 (4) | 319 (4) |  |
| Total | 5,849 | 7,301 | 0.560 |
|  |  |  |  |
| I (%) | 9,275 (79) | 11,620 (80) |  |
| M (%) | 2,423 (21) | 2,982 (20) |  |
| Total | 11,698 | 14,602 | 0.572 |
| **BMI≥ 35** |  |  |  |
| II (%) | 1,110 (60) | 309 (68) |  |
| IM (%) | 654 (36) | 132 (29) |  |
| MM (%) | 78 (4) | 15 (3) |  |
| Total | 1,842 | 456 | 0.005 |
|  |  |  |  |
| I (%) | 2,874 (78) | 750 (82) |  |
| M (%) | 810 (22) | 162 (18) |  |
| Total | 3,684 | 912 | 0.006 |

Abbreviations: PNPLA3, patatin-like phospholipase domain-containing 3; Go-DARTS, Genetics of Diabetes Audit and Research Tayside Scotland; II, individuals with two 148I alleles; MM, individuals with two 148M alleles; IM, heterozygotes; BMI, body mass index.

*Frequencies are in Hardy-Weinberg equilibrium.

†P values were calculated using χ^2^ test with 1d.f. and are two sided.
